# High glycine concentration increases collagen synthesis by articular chondrocytes in vitro: acute glycine deficiency could be an important cause of osteoarthritis

**DOI:** 10.1007/s00726-018-2611-x

**Published:** 2018-07-13

**Authors:** Patricia de Paz-Lugo, José Antonio Lupiáñez, Enrique Meléndez-Hevia

**Affiliations:** 1 Instituto del Metabolismo Celular, Calle Manuel de Falla nº15, La Laguna, 38208 Tenerife, Spain; 2 Present Address: Universidad Internacional de La Rioja, Facultad de Educación, Avenida de la Paz nº137, 26002 Logroño, Spain; 3 Universidad de Granada, Facultad de Ciencias, Departamento de Bioquímica y Biología Molecular I, Avenida Fuente Nueva nº1, 18071 Granada, Spain

**Keywords:** Osteoarthritis, Articular chondrocytes, Cartilage regeneration, Collagen, Glycine

## Abstract

Collagen synthesis is severely diminished in osteoarthritis; thus, enhancing it may help the regeneration of cartilage. This requires large amounts of glycine, proline and lysine. Previous works of our group have shown that glycine is an essential amino acid, which must be present in the diet in large amounts to satisfy the demands for collagen synthesis. Other authors have shown that proline is conditionally essential. In this work we studied the effect of these amino acids on type II collagen synthesis. Bovine articular chondrocytes were cultured under a wide range of different concentrations of glycine, proline and lysine. Chondrocytes were characterized by type II collagen immunocytochemistry of confluence monolayer cultures. Cell growth and viability were assayed by trypan blue dye exclusion method. Type II collagen was measured in the monolayer, every 48 h for 15 days by ELISA. Increase in concentrations of proline and lysine in the culture medium enhances the synthesis of type II collagen at low concentrations, but these effects decay before 1.0 mM. Increase of glycine as of 1.0 mM exceeds these effects and this increase continues more persistently by 60–75%. Since the large effects produced by proline and lysine are within the physiological range, while the effect of glycine corresponds to a much higher range, these results demonstrated a severe glycine deficiency for collagen synthesis. Thus, increasing glycine in the diet may well be a strategy for helping cartilage regeneration by enhancing collagen synthesis, which could contribute to the treatment and prevention of osteoarthritis.

## Introduction

Osteoarthritis is a degenerative joint disease characterized by tensile stiffness, degeneration and progressive loss of articular cartilage. It is one of the most frequent causes of pain, loss of function and disability in adults affecting to up 40% of those aged over 65 (Arden and Nevitt 2006; Bijlsma et al. 2011; Dawson et al. 2004; Mannoni et al. 2003; Zhang et al. 2007). Radiographic evidence of osteoarthritis occurs in the majority of people by 65 years of age and in about 80% of those aged over 75 years (Arden and Nevitt 2006).

Degeneration of the cartilage in osteoarthritis mainly affects the collagen content in the matrix structure (Bank et al. 1997; Dahlberg et al. 2000; Wu et al. 2002). On the other hand, there is also an increase in the synthesis of matrix molecules, including type II collagen (Lohmander et al. 2003; Nelson et al. 1998) and proteoglycans (Rizkalla et al. 1992) in an attempt to preserve the matrix. Degeneration of the matrix affects the mechanical stability of the tissue as well as causes disturbance of chondrocyte function and survival because of the vital chondrocyte–matrix interactions (Buckwalter et al. 2005; Horton et al. 1998; Monfort et al. 2006). In osteoarthritis collagen synthesis is substantially greater than in non-osteoarthritic tissues, but it is not sufficient to compensate for the excessive degradation (Dodge and Poole 1989; Heinegård and Saxne 2011; Lippiello et al. 1997; Nelson et al. 1998; Price et al. 1999; Rizkalla et al. 1992). The progressive imbalance between matrix degradation and its regeneration leads to a marked decrease in the type II collagen content, which eventually results in cartilage damage (Aigner and Stove 2003; Hollander et al. 1995; Nelson et al. 1998; Rizkalla et al. 1992).

The two components of the cartilage (matrix and chondrocytes) are mutually dependent. Chondrocytes deal with matrix synthesis and turnover, and the matrix structure is necessary for the life of chondrocytes. Therefore, degeneration of the cells and the matrix in osteoarthritis is autocatalytic, which causes the disease to progress, like a snake eating its own tail. However, these events do not occur simultaneously. Several studies show that collagen damage is the primary event, while the breakdown of proteoglycans could be a consequence of a poor collagen support (Dodge and Poole 1989; Hollander et al. 1995; Kojima et al. 2001; Poole et al. 2002; Price et al. 1999; Rizkalla et al. 1992; Wu et al. 2002).

Today, there is no cure for this common disease, which for epidemiologists is an enigmatic condition (Arden and Nevitt 2006; Buckwalter and Mankin 1998; Buckwalter et al. 2005; Felson and Nevitt 2004; Heinegård and Saxne 2011). Only symptomatic treatment for pain relief is possible, including pharmacological, non-pharmacological, and surgical approaches (Bijlsma et al. 2011). As Schnitzer (1993) has remarked, the treatment of osteoarthritis has been based entirely on empirical, largely uncontrolled observations, and the focus of therapy has been to relieve pain, the primary symptom of the disease, in hopes of increasing function, such as ability to walk. We think that this sentence summarizes the two main aspects that must be resolved: the lack of a theoretical-formal background, and the need to find a real solution beyond relieving the prejudicial and annoying symptoms.

The regeneration of the cartilage is the main target to solve the problem, and its main key could be the regeneration of collagen (Nelson et al. 1998; Aigner and Stove 2003).

**Fig. 1:**
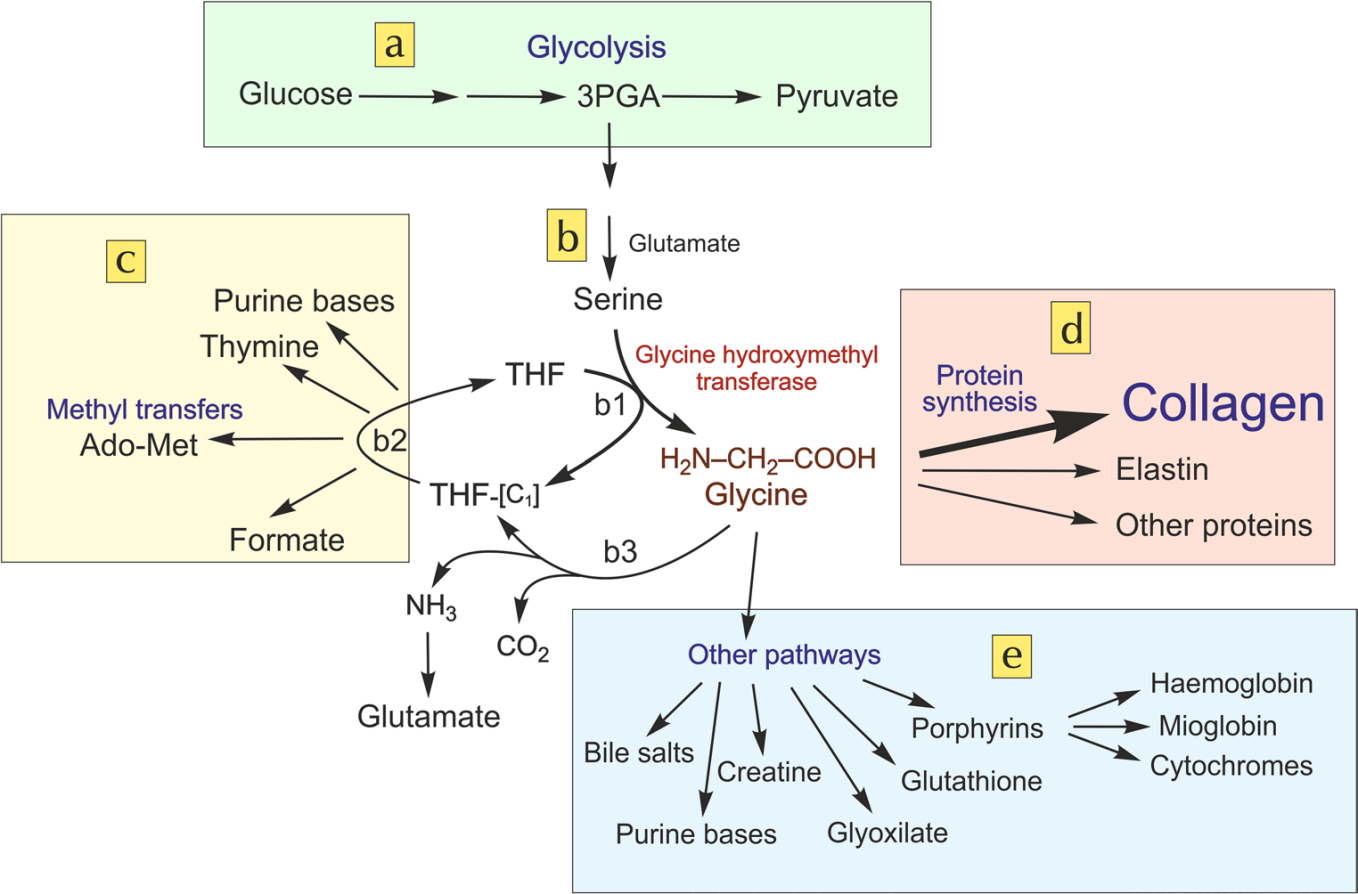
Glycine metabolism pathways. Metabolic pathways involved in the biosynthesis of glycine, and its use for different metabolic functions. **a** Glycolysis, involving the 3-phosphoglycerate (3PGA) metabolite bifurcation. **b** Pathway of serine and glycine synthesis starting from 3PGA. (b1) Reaction of glycine synthesis from serine by glycine hydroxymethyltransferase (EC 2.1.2.1). This is an enzyme-branching break with fixed stoichiometry, which converts serine into glycine with the transfer of a [C_1_] unit to tetrahydrofolate (THF) giving 5,10-methylene tetrahydrofolate (THF-[C_1_]). (b2) Set of reactions that use the [C_1_] unit from THF-[C_1_] regenerating free THF so that it can participate again in reaction b1. (b3) Irreversible reaction of the glycine cleavage system (EC 1.4.4.2), which makes a sink of glycine. **c** Processes involved in THF-[C_1_] metabolism to different products including methyl transfers by adenosyl methionine (Ado-Met). **d** Processes of protein biosynthesis involving glycine, especially the synthesis of collagen. **e** Set of pathways that use glycine as metabolite for other biosynthesis processes. See Meléndez-Hevia and de Paz-Lugo (2008)

Glycine, proline and lysine play a special role in collagen structure (Bowes and Kenten 1948; Pauling and Corey 1951), and their insufficient availability could be a cause to make collagen synthesis and regeneration difficult. Glycine, which is 33% of collagen residues, has been typically classified as a ‘non-essential’ amino acid because human metabolism can synthesize it from serine. However, in previous work (Meléndez-Hevia and de Paz-Lugo 2008) we have shown that the glycine synthesis pathway has a strong stoichiometric restriction that limits its production, independently of the capacity and the regulatory mechanisms of the enzymes, as shown in Fig. 1.

The biosynthesis of glycine from serine occurs in a reaction that does not establish a bifurcation metabolite from a common intermediate towards two alternative products whose fluxes can be distributed independently; rather it is an enzyme-branching break with fixed stoichiometry yielding two different products: glycine, and the [C_1_] unit carried by tetrahydrofolate. This determines that the production of either of them is conditioned by the consumption of the other because both have to be consumed in equal quantity. This constraint between the two independent pathway series can cause a lack of one of them because the product that is less used will determine a mathematical limit on the production of the second. No previous case of such a stoichiometric constraint that can lead to a deficiency of any product has been described in the metabolic network. We have called it (and other similar cases that may eventually be discovered in the future) weak links in metabolism (Meléndez-Hevia and de Paz-Lugo 2008).

On the other hand, the synthesis of collagen microfibril is a very complex process where a high fraction (30–90%, depending on the tissues and the age of individuals) of the newly synthesized collagen is degraded in the procollagen cycle within minutes of its synthesis to achieve the correct triple helix folding (see Meléndez-Hevia et al. 2009 and references therein). This greatly increases the need for glycine since most of it, which comes from procollagen degradation in the cycle, is not available for reuse (Gibson et al. 2002). Hydroxyproline and hydroxylysine cannot be recycled because they must be incorporated in the procollagen peptide as proline and lysine, respectively, thereby greatly increasing the needs for these amino acids as well.

Our results reported in a subsequent work (Meléndez-Hevia et al. 2009) showed that the use of the [C_1_] unit is much lower than the need for glycine, especially for collagen synthesis. Thus, glycine must be considered an *essential* amino acid because the capacity of its synthesis is much lower than its actual need. We also showed that this deficiency is not covered with a regular diet so glycine should be added to it as a nutritional supplement in high amounts, about 10 g/day. A review of the data on this subject corroborates the need for glycine in the diet (Wang et al. 2013). Wu et al. (2011) have shown that proline it is conditionally essential under some conditions, which means that the capacity of its synthesis could not sufficiently account for its need. Finally, lysine is an essential amino acid, whose availability depends entirely on the diet.

Proteoglycans have no special abundant amino acid such as glycine or proline in collagen; see, e.g., Uldbjerg et al. (1983). Moreover, glucosamine and any sugar derivatives, such as galactosamine, main components and precursor of proteoglycans do not have a problem of design in its synthesis pathway like glycine has. Mankin et al. (1981) demonstrated that the use of glucosamine was the same in normal and osteoarthritic samples. Results reported by Qu et al. (2006) showed that glucosamine and other similar sugars have no effect on the synthesis of proteoglycans by chondrocytes in vitro. Roman-Blas et al. (2017) confirmed the ineffectiveness of glucosamine and chondroitin sulfate showing that the placebo had even better effect that these products.

Thus inefficient collagen synthesis by chondrocytes to renew the old degraded molecules may be a main cause of osteoarthritis, in agreement with Aigner and Stove (2003). Rizkalla et al. (1992) pointed out that “if collagen damage could be prevented and its repair promoted then it may be possible for cartilage to repair itself”.

Therefore, according to the reasoning presented above, we can assume that a cause of osteoarthritis could be a poor availability of some essential amino acids to build collagen. Thus, collagen synthesis could be improved if chondrocyte metabolism had the amino acids mentioned (especially glycine) available in the necessary quantities. In this work, we have studied the effect of glycine, proline and lysine on the synthesis of type II collagen in articular chondrocytes cultured in vitro. Our experimental results agree with the theoretical predictions.

## Materials and methods

### Chemicals

Hyaluronidase type V, pancreatic elastase, collagenase type IV (*Clostridium histolyticum*), penicillin-G, Sigma-Aldrich Ham’s F12 medium (Sigma-Aldrich 2017), fetal bovine serum (FBS), bovine serum albumin (BSA), Tris, Hepes, trypan blue, and Giemsa stain solution were obtained from Sigma (USA); pepsin, pronase (*Streptomyces griseus*) and streptomycin sulfate were obtained by Fluka (USA); trypsin was obtained from Gibco (USA); mouse monoclonal primary antibody directed against bovine type II collagen was obtained from Labvision Corporation (USA); FITC conjugated secondary antibody (sheep anti mouse IgG conjugated to fluorescein isothiocyanate) was obtained from Stressgen Biotechnologies Corporation (USA); Glycine, *l*-proline, *l*-lysine, *l*-isoleucine and *l*-aspartic acid were Pharma grade, obtained from Quimipur (Spain); all other chemicals were of reagent grade and were purchased from Sigma, Quimipur, and Panreac (Spain).

### Culture media and reagents

Basal culture medium (BCM)—Ham’s F12 medium containing 10% fetal bovine serum (FBS), 100 U/mL penicillin and 100 µg/mL streptomycin. Hank’s buffered salt solution (HBSS) (Hanks 1976)—prepared in our laboratory at pH 7.2 containing 100 U/mL penicillin and 100 *µ*g/mL streptomycin. Phosphate-buffered saline (PBS)—Dulbecco’s phosphate-buffered saline at pH 7.2. Concentrated Tris buffered saline (TBS10)—1 M Tris, 2.0 M NaCl, 50 mM CaCl_2_, pH 7.9. Diluted Tris buffered saline (TBS1)—100 mM TRIS, 200 mM NaCl, 5.0 mM CaCl_2_, pH 8.0. Pepsin solution (PepS)—10 mg pepsin/mL dissolved in 0.05 M acetic acid. Pancreatic elastase solution (PES)—1 mg elastase/mL dissolved in TBS1. Trypsin solution—250 mg trypsin, 20 mg EDTA per 100 mL PBS, pH 7.5.

### Isolation and cultures of chondrocytes

Isolation and culture of chondrocytes were performed by a modified protocol, according to previously described methods (Oesser and Seifert 2003; Qu et al. 2006). Cartilage samples were dissected aseptically from the metacarpophalangeal joints of 12- to 18-month-old steers obtained from a local abattoir within 2 h after slaughter. Shavings of hyaline cartilage (3 × 3×1 mm) were removed from the outer two-thirds of the articular cartilage, such that contamination with bone cells or other connective tissue cells could be avoided. Cartilage slices were minced and extensively washed with HBSS. The cartilage was diced aseptically into ≈ 1-mm^3^ pieces and subsequently digested with 2000 U/mL hyaluronidase and 30 U/mL pronase in HBSS for 60 min at 37 °C. Then, cartilage fragments were washed twice in HBSS and transferred into a collagenase solution (8 U/mL) in BCM at 37 °C for 7 h with gentle shaking. During this 7 h, collagen network was digested and cells were liberated to the culture medium. Fibroblasts do not resist this long treatment with collagenase, so we have avoided their potential contamination in the cultures. The resulting cell suspension was filtered through a 20-µm nylon mesh and centrifuged at 1000 rpm for 10 min. After centrifugation the supernatant was discarded and the pellet resuspended in 5 mL of BCM.

The vital chondrocytes extracted in the final cell suspension were counted by the trypan blue exclusion method in a Neubauer chamber. Cells were seeded in 24-well plates at a density of 150,000 cells/mL and cultured at 37 °C in the basal medium (BCM) under an atmosphere of 5% O_2_, 5% CO_2_ and 90% N_2_ with 85% humidity. The culture medium was changed every 48 h for the duration of the experiments. No subculture cells were used to avoid loss of their differentiated type II collagen phenotype (Cao et al. 1997). All operations with cells (isolation, seeding and changes of culture medium) were carried out in a Telstar PV-100 vertical laminar flow bench. Cell cultures were carried out in a benchtop CO_2_ 8 IR incubator (New Brunswick Scientific). At 24 h after cells seeding, when they become attached and evenly distributed in the wells, glycine, *l*-proline, *l*-lysine, *l*-isoleucine, or *l*-aspartic acid were added to the wells at appropriate concentrations according to each experiment in the range of 0.25–7.0 mM. Cells without amino acid addition were used as the control group. All the cells in the experimental and control groups were incubated for 15 days from seeding, analyzing cell shape, growth, viability, and type II collagen every 48 h. All experiments were made from three different cows on different days, taking duplicate samples in each.

### Cell shape, growth and viability

Chondrocyte shape in the culture monolayers was checked by Giemsa stain. The cells were isolated from the monolayers by rinsing with PBS to remove traces of serum and incubated with 250 µL of trypsin solution for 2 min at room temperature. After eliminating the excess of trypsin, leaving a few drops over the monolayer, cells were incubated at 37 °C for 15 min in the CO_2_ incubator. Then, 1 mL of BCM was added to the cell layer and cells were recovered. Number of total cells and cell viability were assessed by trypan blue dye exclusion method with the Vi-CELL XR cell viability analyzer (Beckman Coulter, California, USA).

### Immunocytochemistry of monolayer cultures

Chondrocytes were seeded on gelatinized glass coverslips at a density of 150,000 cells/mL and cultured as described above until reaching confluence. Then the cells were fixed with 2% (w/v) paraformaldehyde for 30 min at room temperature. After three washes with PBS, autofluorescence was eliminated with sodium borohydride 1 mg/mL for 10 min. Then, after rinsing in PBS, cells were permeabilized using 0.2% Triton X-100 in PBS for 10 min and endogenous peroxidase activity was blocked with 3% (w/v) BSA in PBS/0.1% Triton X-100 for 10 min. The chondrocytes were rinsed again three times in PBS and incubated for 1 h with mouse monoclonal primary antibody directed against bovine type II collagen (Labvision) diluted at 3% (w/v) in BSA/PBS. After washing again in PBS, the cells were incubated for 30 min with the FITC conjugated secondary antibody (Stressgen) diluted at 3% (w/v) in BSA/PBS. The samples were finally mounted on Fluoromount-G fluorescent assembly (SouthernBiotech, Alabama, USA) and examined under a Leica DM IL inverted microscope with digital camera, phase contrast and fluorescence equipment. As negative control, the primary antibody was replaced by 3% (w/v) BSA in PBS. Cells/matrix proportion volume was estimated using the CorelDraw Graphics Suite X7 software (Corel Corporation, Canada).

### Quantification of type II collagen

Type II collagen was assayed in the monolayer, where the chondrocytes are embedded in the matrix, every 48 h for 15 days after amino acid additions. Bovine type II collagen in the cell monolayers was determined by means of the Arthrogen-CIA type II collagen capture ELISA kit (Chondrex, Inc., MD Biosciences, Zurich). To solubilize the newly synthesized collagen, the cell culture medium was removed and the cell layers were washed with HBSS. After removing the HBSS, 250 µL-0.05 M acetic acid was added to each well; cells were dispersed with careful scraping, and the cell suspension of each well was transferred to a microcentrifuge tube and were incubated with 25 µL-pepsin solution (PepS) for 24 h at 4 °C. Subsequently, 25 µL TBS10 were added to stop the reaction, and then 2.5 µL NaOH 6 N are added to adjust pH. The remaining polymeric collagen was monomerized by adding 25 µL of pancreatic elastase solution (PES) to each tube and incubated for 24 h at 4 °C with gentle shaking. Then the tubes were centrifuged at 10,000 rpm for 5 min, and aliquots of the supernatant were taken for type II collagen assay by ELISA method with the Chondrex Type II Collagen Detection Kit, following the assay protocol described by the manufacturer (Chondrex, Inc. 2016) using a Greiner high-binding multiwell 96 plates. Artwork (Figs. 1, 4, 5, 6) was created with Corel DRAW Graphics Suite X7.

### Statistical analysis

All the quantitative values were obtained from three experiments with three different cows, analyzing two or three samples in each one, by extracting the samples from each monolayer in the culture. All coefficients of variation *V* = (*s*/*X*) × 100 calculated for each group of the two or three assays of each sample (controls and each addition of amino acids) were between 4.5 and 5.7, which mean low standard error in the assay procedure. The results presented are the mean, with the error bars (SD) of the three different samples. The significance of differences between treated groups and non-treated control were assessed by an ANOVA test, followed by the Tukey test for multiple comparisons using GraphPad Prism 4 software (Motulsky 2003). Differences with *P* < 0.05 were considered to be statistically significant.

## Results

### Chondrocytes cultures

**Fig. 2:**
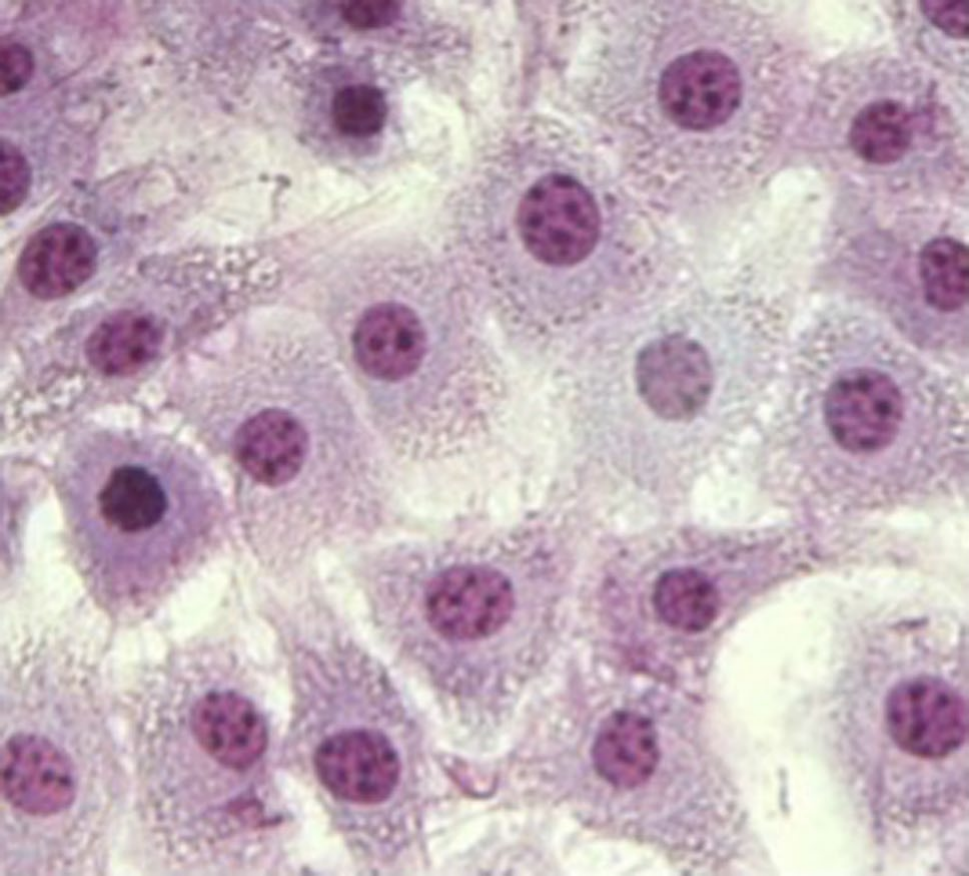
Bovine chondrocytes in the well plate monolayer (40× objective). Cells were fixed and stained after 7 days of culture with Giemsa. The cultured cells look normal showing their typical pyramidal shape.

Giemsa stain of cells was made to check chondrocyte morphology. Figure 2 shows a typical result where chondrocytes have their normal polygonal shape.

**Fig. 3:**
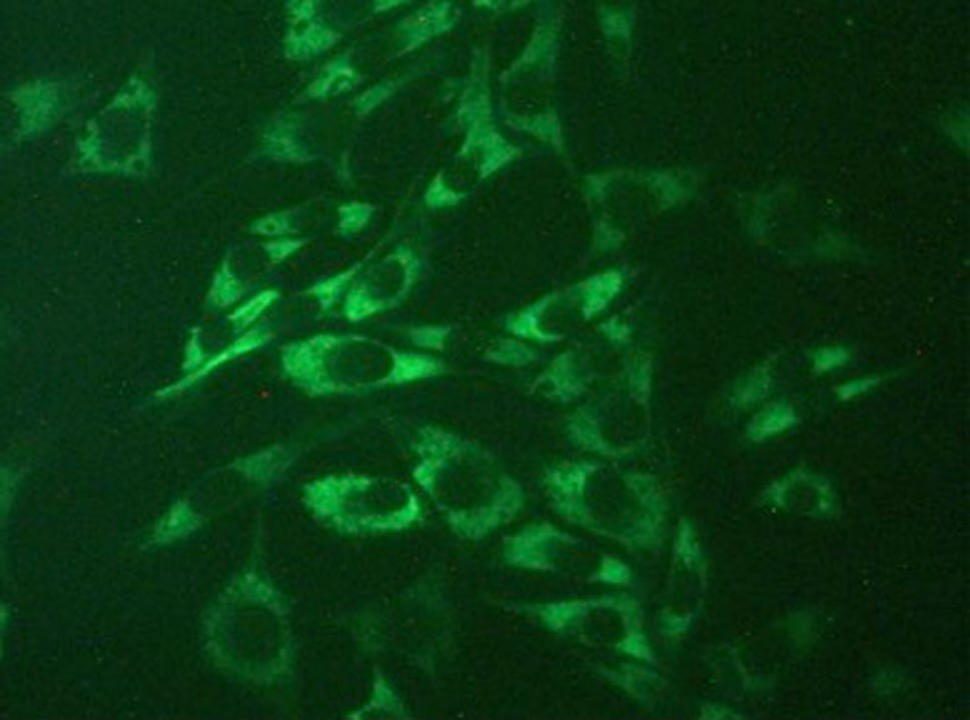
Immunofluorescence staining of type II collagen in cultured chondrocytes (40× objective) in monolayer. Typical result of the monolayer culture with glycine enriched basal medium. Cells were fixed and stained after 7 days of culture with specific antibodies against type II collagen. The picture demonstrates that cultured cells are indeed chondrocytes because, apart from their typical shape, they synthesize type II collagen. The picture shows only the dyed type II collagen, and the cells contours are seen by contrast. Similar results were obtained after treatment with other amino acids in the culture medium

In order to check the validity of the experimental model, we assayed in the monolayer type II collagen by specific immunofluorescence stain; a typical result is shown in Fig. 3.

The close relationship remarked above between cells shape and survival, and matrix structure, demonstrates a good development of the sample in the monolayer experimental model. According to Kuettner et al. (1982), chondrocytes begin to resynthesize pericellular matrix after becoming anchored to the plastic surface, which is fully established after 3–4 days in culture and is morphologically indistinguishable from that of the normal chondrocytes in vivo. The results shown in Fig. 3 agree with this statement. Trypan blue exclusion stain showed that supplementing the culture medium with any amount of amino acids used in our experiments did not affect the viability of the cells. All cultures were between 80 and 89% viability with no significant differences among them for each amino acid concentration, nor was there any significant change of viability during the 15 days of culture after 2 days necessary for monolayer stabilization, both in controls and in cultures with amino acids added at any concentration. Standard deviations were the same range in all cultures, all of them between 3.28 and 8.97.

### Effect of amino acids on collagen synthesis

**Fig. 4:**
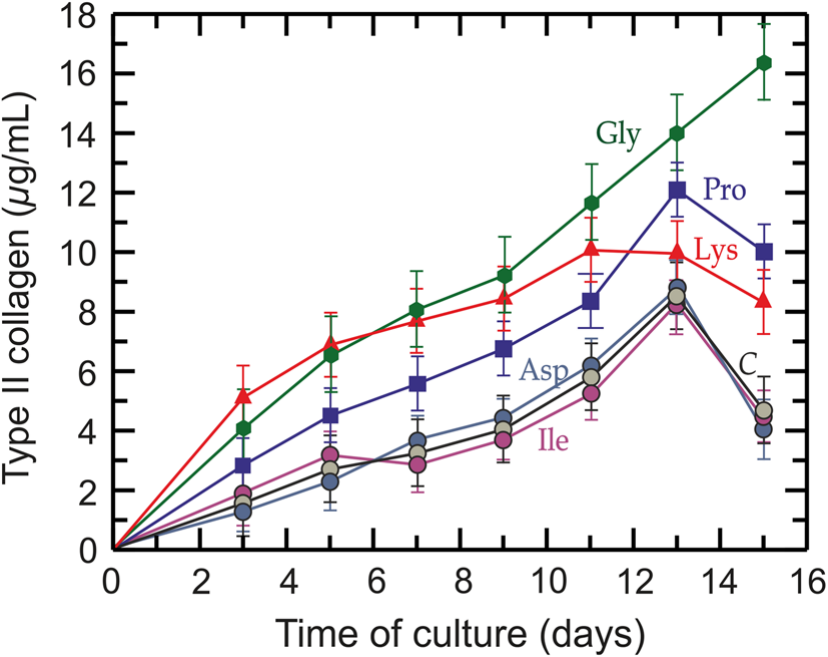
Progress in collagen synthesis by articular chondrocytes. Specific effect of each amino acid 1.5 mM on type II collagen production by chondrocytes in the monolayer during culture development. Green hexagon: glycine (green line); blue square: *l*-proline (blue line); red rectangle: *l*-lysine (red line); dark blue circle: *l*-aspartic acid (dark blue line); purple circle: *l*-isoleucine (purple line); gray circle: control (*C*) (black line)

Figure 4 shows the progress of type II collagen synthesis during the 15 days of tissue culture. As can be seen, in the basic control conditions (gly 0.25 mM, pro 0.15 mM, lys 0.5 mM) there is an increase in type II collagen synthesis until day 13 and the synthesis decays thereafter. Addition of the three amino acids to the medium to reach 1.5 mM produces an increase in the synthesis, but the profile effects of proline and lysine are similar to the control, while the effect of glycine is greater than all of them and does not decay at the end of the culture. Addition of *l*-isoleucine and *l*-aspartic acid (used as control amino acids) to the medium at the same concentrations did not produce any significant effect on collagen production different from the basic control.

**Fig. 5:**
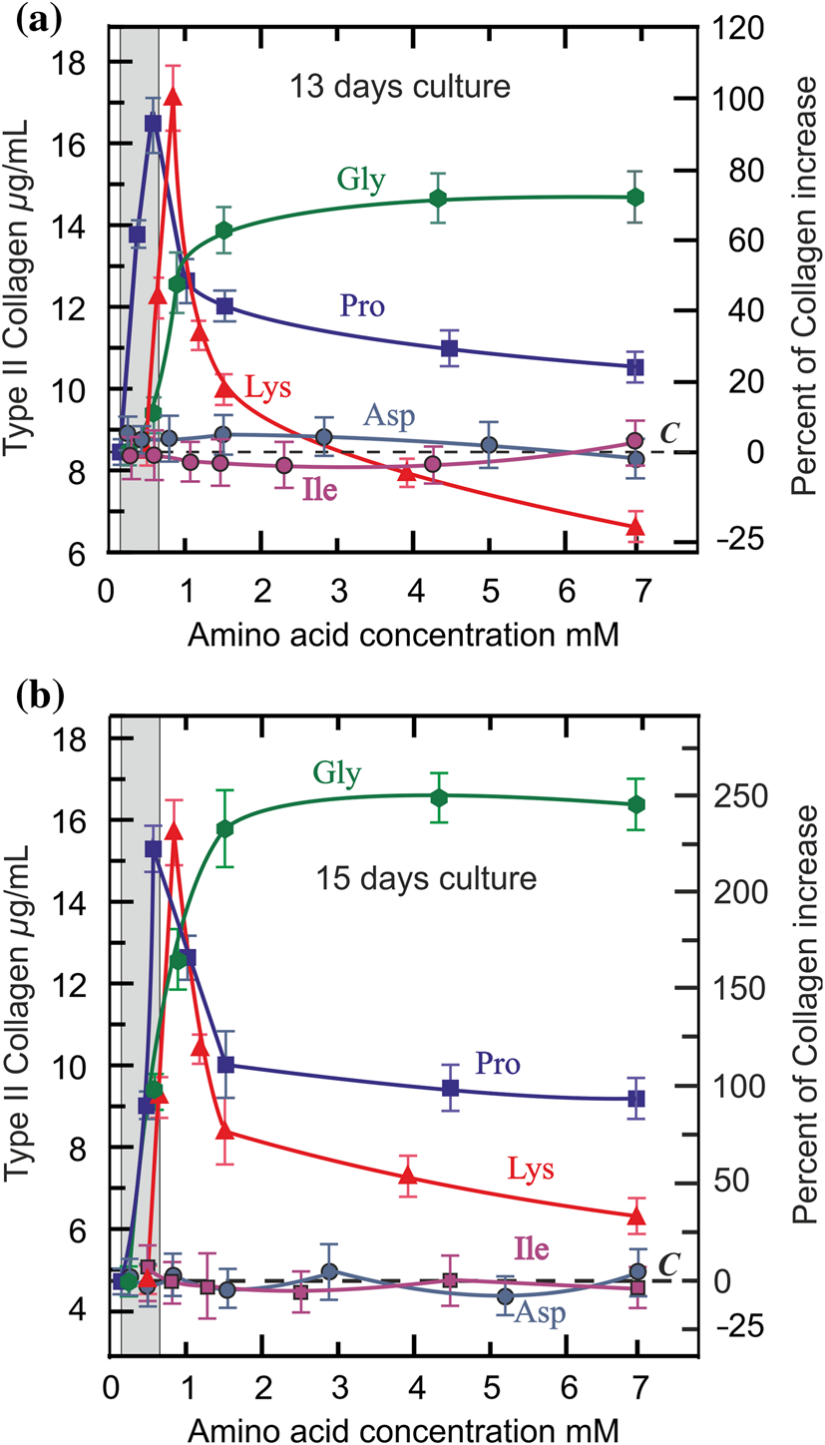
Effect of each amino acid concentrations on type II collagen synthesis. Type II collagen production by chondrocytes in the monolayer at 13 days (**a**) and 15 days (**b**) of culture development under each amino acid concentration: green hexagon: glycine (green line); blue square: *l*-proline (blue line); red rectangle: *l*-lysine (red line); dark blue circle: *l*-aspartic acid (dark blue line); purple circle: *l*-isoleucine (purple line). The dotted line (*C*) means the collagen production under control conditions in the regular medium (gly 0.25 mM, pro 0.15 mM, lys 0.5 mM). The area marked in gray to the left of the graph shows the normal range of concentrations of these amino acids in the plasma (Felig and Wahrent 1971; Goodwin 1968; Javitt et al. 2001; Nakazawa et al. 1982; Summer and Roszel 1965)

The increase of type II collagen in the monolayer was different according to each amino acid concentration in the medium. Figure 5 shows the results at 13 and 15 days of culture.

Results in Fig. 5b, at 15 days culture, show that the need of glycine is the highest, having to be 1.5 mM to reach about 225% collagen increase over the control. The same effects were achieved with proline and lysine, but their need was lower, up to 0.6 and 0.8 mM, respectively. However, the most important feature of these results is that glycine maintains its stimulating effect at higher concentrations (up to 7 mM), while the increased concentrations of proline and lysine declined their effects, their being at 1.5 mM one half and one-third, respectively, of the effect achieved by glycine. Increased concentrations of *l*-aspartic acid or *l*-isoleucine in the medium up to 7.0 mM did not produce any effect, which demonstrates the specific need for glycine, proline and lysine.

### Effect of amino acids on cell growth

**Fig. 6:**
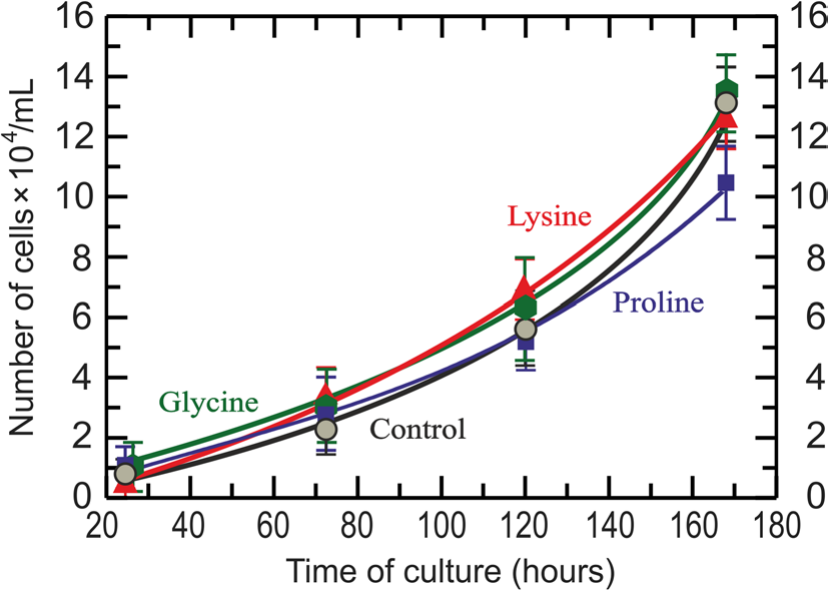
Effect of amino acids on chondrocyte growth. Growth curves of chondrocytes under amino acid concentrations that provoked the highest type II collagen synthesis, according to data shown in Fig. 5(a) and (b). Green hexagon: glycine, 6.90 mM (green line); blue square: *l*-proline, 0.60 mM (blue line); red rectangle: *l*-lysine, 0.85 mM (red line); gray circle: control (black line)

Increased concentrations of glycine, proline or lysine in the assayed ranges that promote increase in collagen synthesis did not make changes in cell growth. This effect was seen for all concentrations of amino acids assayed. Figure 6 shows the results at concentration of each amino acid that promotes the highest collagen synthesis (7.0 mM glycine, 0.6 mM *l*-proline, 0.8 mM *l*-lysine); *l*-aspartic acid and *l*-isoleucine (not shown) did not affect cell growth significantly.

## Discussion

Results presented in Figs. 4 and 5 show that increased concentrations of glycine, proline and lysine in the basal medium enhance type II collagen synthesis. The effects of these amino acids are independent as they are *independent variables* because the degradation of any one of them does not give rise to a specific metabolite for the synthesis of any other. These results suggest that an increase in the concentration of these amino acids could improve the regeneration of the articular cartilage matrix.

As seen in Fig. 5, proline and lysine produce a great effect at low concentration, but this effect decreases from 0.6 mM and 0.85 mM, respectively, while the effect of glycine, although lesser at low concentration, exceeds the previous ones from ≈ 1.0 mM and, unlike the previous ones, this increase continues more persistently even up to higher concentrations. The greater effects produced by proline and lysine are within their physiological concentration in plasma, while the effect of glycine corresponds to a much higher range, demonstrating a severe generalized glycine deficiency for synthesizing collagen. The normal plasma concentration of glycine is 0.25-0.5 mM (Felig and Wahrent 1971; Javitt et al. 2001), which corresponds to a normal intake of 1.50-3.00 g/day (Gibson et al. 2002). According to the results reported by Javitt et al. (2001), an increase in the diet of 10 g/day could increase the plasmatic concentration between 3 and 4 times up to 1–2 mM, which means an increase about 200% in collagen synthesis (Fig. 5b).

An important key to understanding these results is the fact that glycine, the most necessary amino acid for collagen synthesis, is highly essential so it must necessarily be incorporated into the diet as a nutritional supplement; this need is generalized in all animals from ≈ 30 kg of body mass and increases with body weight. In a 70-kg human being, this glycine deficiency is ≈ 10 g daily—probably the highest of the essential amino acids, according to our previous results (Meléndez-Hevia et al. 2009); see also (Gibson et al. 2002).

Several works have shown that collagen hydrolysate could enhance the biosynthesis of type II collagen by chondrocytes in vitro and may be a way to treat osteoarthritis (Ameye and Chee 2006; Bello and Oesser 2006; McAlindon et al. 2011; Moskowitz 2000; Oesser et al. 1999). Collagen hydrolysate has 33% glycine residues (25% of its mass), so 10 g of hydrolysate (the daily dose used) means 2.5 g of glycine, which is insufficient for the metabolic needs. It may produce a slight improvement, but better results could be achieved with a daily dose of 10 g of glycine which we are proposing here. On the other hand, gelatin or collagen hydrolysate are not advantageous as proline or lysine sources because their hydroxylated forms, which means about 30-50% of these amino acid residues (Barnes et al. 1974), are useless for reutilization. Thus, although results with collagen hydrolysate may be moderate, they are an indication of the glycine needed to propitiate cartilage regeneration.

Therefore, increasing glycine in the diet could be a possible way of contributing to fight and prevention of osteoarthritis to improve cartilage regeneration by means of enhancing collagen synthesis. It may be that the deficiencies mentioned above are not the only cause, but it is certainly a feasible place to start. To this end our results suggest a viable strategy through increasing the amounts of these amino acids (glycine especially) in the diet. As these deficiencies will obviously affect other connective or mechanical tissues, such as bones, tendons, ligaments and skin, we would like to remark that this conclusion might also well be applied in the treatment of these damaged tissues in conditions such as osteoporosis.
